# Generation and verification of QFix kVue Calypso‐compatible couch top model for a dedicated stereotactic linear accelerator with FFF beams

**DOI:** 10.1120/jacmp.v16i4.5441

**Published:** 2015-07-08

**Authors:** Stephen J. Gardner, Misbah Gulam, Kwang Song, Haisen Li, Yimei Huang, Bo Zhao, Yujiao Qin, Karen Snyder, Jinkoo Kim, James Gordon, Indrin J. Chetty, Ning Wen

**Affiliations:** ^1^ Department of Radiation Oncology Henry Ford Health System Detroit MI; ^2^ Department of Radiation Oncology 21st Century Oncology Farmington Hills MI USA

**Keywords:** couch modeling, VMAT delivery, SRS/SBRT delivery, dose delivery uncertainties

## Abstract

This study details the generation, verification, and implementation of a treatment planning system (TPS) couch top model for patient support system used in conjunction with a dedicated stereotactic linear accelerator. Couch top model was created within the TPS using CT simulation images of the kVue Calpyso‐compatible couchtop (with rails). Verification measurements were compared to TPS dose prediction for different energies (6 MV FFF and 10 MV FFF) and rail configurations (rails in and rails out) using: 1) central axis point‐dose measurements with pinpoint chamber in water‐equivalent phantom at 42 gantry angles for various field sizes ($2\times 2\;{\text{cm}}^{2},4\times 4\;{\text{cm}}^{2},10\times 10\;{\text{cm}}^{2}$); and 2) Gafchromic EBT3 film parallel to beam in acrylic slab to assess changes in surface and percent depth doses in PA geometry. To assess sensitivity of delivered dose to variations in patient lateral position, measurements at central axis using the pinpoint chamber geometry were taken at lateral couch displacements of 2, 5, and 10 mm for 6 MV FFF. The maximum percent difference for point‐dose measurements was 3.24% (6 MV FFF) and 2.30% (10 MV FFF). The average percent difference for point‐dose measurements was less than 1.10% for all beam energies and rail geometries. The maximum percent difference between calculated and measured dose can be as large as 13.0% if no couch model is used for dose calculation. The presence of the couch structures also impacts surface dose and PDD, which was evaluated with Gafchromic film measurements. The upstream shift in the depth of dose maximum (dmax) was found to be 10.5 mm for 6 MV FFF and 5.5 mm for 10 MV FFF for ‘Rails In’ configuration. Transmission of the treatment beam through the couch results in an increase in surface dose (absolute percentage) of approximately 50% for both photon energies (6 MV FFF and 10 MV FFF). The largest sensitivity to lateral shifts occurred at the lateral boundary of the rail structures. The mean magnitude (standard deviation) of the deviation between shifted and centered measurements over all field sizes tested was 0.61% (0.61%) for 2 mm shifts, 0.46% (0.67%) for 5 mm shifts, and 0.86% (1.46%) for 10 mm shifts.

PACS numbers: 87.56.‐v, 87.56.Da, 87.56.Fc

## I. INTRODUCTION

The treatment of stereotactic radiosurgery (SRS) and stereotactic body radiation therapy (SBRT) targets requires the incorporation of many advancements within the linear accelerator. These advancements can include flattening filter‐free (FFF) technology^1^, ^2^ to achieve high dose rates, image‐guidance capabilities (including MV,^3^, ^4^ kV,^(5)^ and CBCT^(6)^ imaging), high‐definition MLC widths for enhanced beam shaping,^7^, ^8^ abilities for real‐time tumor tracking,^(9)^ motion management,^10^, ^11^ and a patient positioning system^12^, ^13^ to enable submillimeter precision in target localization.

The patient positioning system includes any immobilization devices utilized, as well as the treatment couch, which provides rigid support for the patient during treatment. Treatment plans that do not account for a couch structure could involve significant discrepancies between the planned and actual delivered dose. Several studies have found that neglecting the couch in the dose calculation leads to mischaracterization of the delivered dose to the patient, and the impact of couch attenuation should be carefully evaluated.^14^, ^15^, ^16^, ^17^, ^18^, ^19^ Additionally, the recently‐published AAPM TG 176 report is available to provide guidance to physicists for modeling a couch top for accurate and realistic dose calculation.^(20)^

The Edge linear accelerator (Varian Medical Systems, Palo Alto, CA) is a dedicated stereotactic suite involving multiple localization modalities and was recently installed in our clinic. The Edge includes multiple target localization systems: the Calypso system (Calypso Medical Technologies, Inc., Seattle, WA) for electromagnetic transponder tracking, Optical Surface Monitoring System (OSMS) for real‐time surface tracing, and on‐board imaging (OBI) for planar and CBCT imaging. To allow for the use of the Calypso system, a nonconductive couch top with rails was installed (QFix kVue Calypso‐compatible couch top) (QFix, Avondale, PA). Though the kVue couch top is compatible with all of the localization systems offered on the Edge linac, there is currently no commercial TPS couch top model available for Eclipse v. 11 (Varian Medical Systems).

This study details the creation, verification, and implementation of the kVue couch top TPS model in our clinic. The creation of the couch top model, including the CT scan of the couch top, electron density determination, and contouring of each component, is described in the Materials & Methods section. The verification of the couch top is also discussed in the Methods section. The clinical implementation is discussed in the Discussion section. These tests were designed to be easily replicated for modeling of various types of couch tops in a variety of clinical settings.

## II. MATERIALS AND METHODS

**Figure 1:**
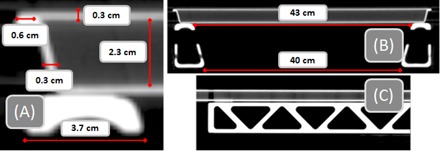
Zoomed‐in view (a) of the couch top during CT simulation with relevant dimensions of the flat‐panel portion of the couch. Transverse view (b) of the couch top; the rails are in ‘Rails Out’ configuration. Sagittal view (c) of the couch rail structure (note the periodic structure of the rails).

**Figure 2:**
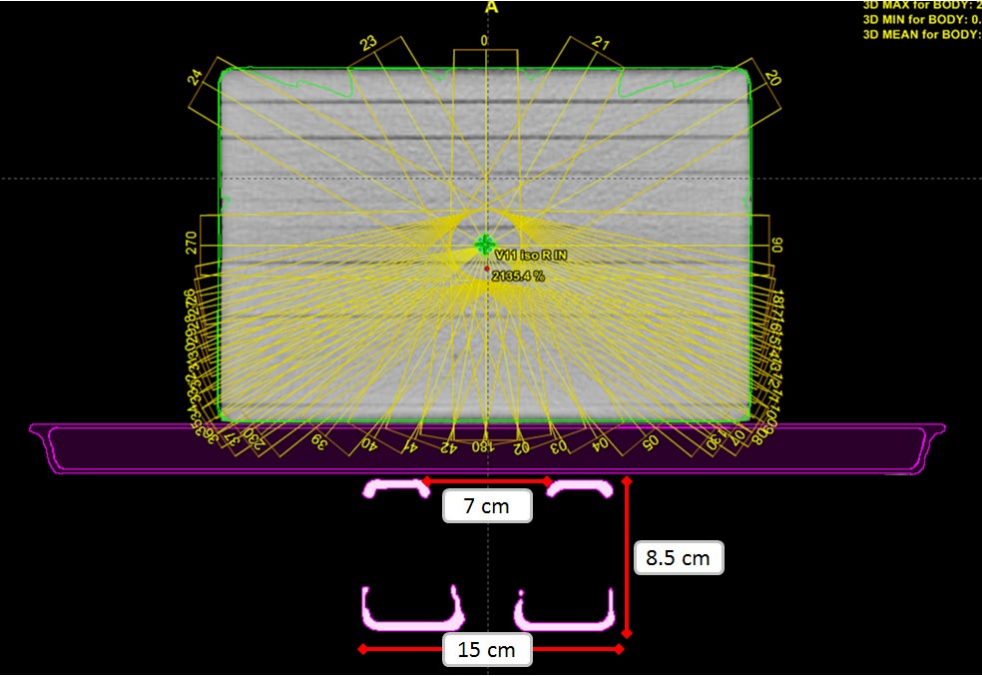
Transverse view of the TPS couch model with ‘Rails In’ configuration. Note the distinct elements of the couch model: couch surface (exterior), couch interior, and couch rails. Also shown in this figure are the water‐equivalent slab phantom used for chamber measurements and the 42 gantry angles used for chamber measurements.

The QFix kVue Calypso‐compatible couch top was recently installed in our clinic as part of the Varian Edge radiosurgery system. This couch top utilizes a support structure composed of nonconductive composites and a rail system. The couch top support structure measures 132.5 cm (longitudinal length), 51.4 cm (lateral width), and 2.8 cm (couch thickness). The couch top support structure surface is composed of high‐strength fiber composites that are completely free of metal and nonconductive. The rail system utilizes a periodic lattice structure and is designed to minimize imaging artifacts that may occur with traditional rails.^(21)^ The rail structures extend 40 cm beyond the couch base and measure 9 cm in height and 6 cm in lateral width at the widest point. With ‘Rails Out’ configuration, the rails span a lateral distance of 51 cm (see Fig. 1 for dimensions of couch top and rails components). With ‘Rails In’ configuration, the rails span a lateral distance of 15 cm (see Fig. 2 for further dimensions with ‘Rails In’ configuration). The following sections describe the process of implementing and validating the couch model: the simulation CT of the couch, TPS couch model creation, point‐dose chamber verification, film verification, and sensitivity test.

### A. CT scan of couch top

The couch top and rails were scanned using simulation CT in helical mode (Philips Brilliance Big Bore; Philips Healthcare, Andover, MA) in a variety of configurations (see Fig. 1 for view of ‘Rails Out’ configuration during CT simulation). The configurations were chosen to cover all clinically appropriate rail positions: both rails in, both rails out, right rail in, and left rail in. The relevant scan parameters utilized include: 650 mm FOV, 1.0 mm slice thickness, 125 kVp.

### B. Incorporation of couch top into TPS

The CT image data was imported into Eclipse v. 11. The couch structure model is composed of three distinct components: 1) couch surface, which is composed of composite Kevlar material; 2) couch interior, which contains low‐density foam within the couch surface structure; and 3) couch rails. See Figure 2 for view of couch model in TPS. To generate the couch model, two characteristics of the components must be determined: the physical dimensions/location of the component, and the material composition of the component. In the couch model, a three‐dimensional contour is used to denote the physical extent of each component, while the HU value is used as a surrogate for the material composition. Automated contouring was performed using HU threshold‐based tool to delineate the couch surface and rail structures. The couch interior structure was defined as the material within the couch surface structure. To determine the HU values of each component, intensity profiles were generated through each component at several longitudinal positions. The mean HU value (rounded to the nearest ten HUs) was then used as the default HU value for the corresponding couch model structure.

In the creation of the couch model, the thickness of the couch interior, surface, and support rails was dependent on the HU threshold used for autocontouring of each component. To further optimize the model, the thicknesses of each component were tweaked to better match physical measurements of couch component dimensions. This was done prior to couch verification measurements.

Thus, a TPS couch model was generated, which consisted of contours of the couch interior, couch surface, and couch support rails. These contours were assigned an HU value (which corresponds to an electron density) and the couch structure set was saved to the TPS database. To use the couch model in a treatment plan, the couch structure set must be transferred to the treatment plan of interest and registered to the CT images used for planning.

### C. Couch model verification

Two types of measurements were acquired for couch model validation. These measurements were designed to comply with the recommendations of TG‐176. For attenuation verification, point‐dose measurements were acquired in water‐equivalent slab phantom (30 cm×30 cm×20 cm thick) with pinpoint chamber (PTW TN 31016, PTW, Freiburg, Germany; collection volume 0.015 cm^3^). Measurements were performed for the two available machine energies (6 MV FFF and 10 MV FFF), three field sizes ($2\times 2\;{\text{cm}}^{2},4\times 4\;{\text{cm}}^{2}$, and $10\times 10\;{\text{cm}}^{2}$), and two couch rails configurations (‘Rails In’ and ‘Rails Out’). The maximum dose rates were used during these tests: 1400 MU/min for 6 MV FFF and 2400 MU/min for 10 MV FFF. The field sizes were chosen to reflect the intended use of this machine (namely, smaller fields for cranial and extracranial stereotactic treatments). A total of 42 gantry angles were used for point‐dose measurements, with gantry angle resolution between consecutive measurements divided into three zones (see Table 1): Zone 1 — posterior entry through the couch top (angular resolution of 10°/measurement); Zone 2 — oblique entry through the couch top and/or rails (angular resolution of 2.5°/measurement); and Zone 3 — no transmission through the couch and/or rails (angular resolution of 30°/measurement). See Fig. 2 for TPS view of the beam angles and water‐equivalent slab phantom used in this study. The delivery for these measurements was automated using XML scripting and TrueBeam (Varian Medical Systems) developer mode. A script was written for each energy and field size combination, and 100 MU were delivered for each gantry angle. To determine the absolute dose calibration factor for the chamber measurements, an AP calibration field was delivered for each field size and energy tested.

**Figure 3:**
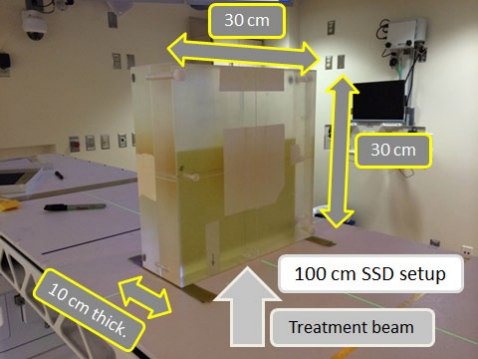
Experimental setup for film dosimetric measurements. Gafchromic EBT3 film was placed in BrainLAB acrylic phantom (10 cm thickness) and aligned at isocenter. All films were irradiated at 100 cm SSD. This figure shows PA delivery with the treatment beam transmitted through the couch top. Baseline measurements with AP delivery were also acquired.

To verify the modeling of surface dose and percent depth dose (PDD), film measurements were acquired with GafChromic EBT3 film (International Specialty Products, Wayne, NJ). All film measurements were acquired using BrainLAB acrylic slab phantom (BrainLAB, Feldkirchen, Germany), which has dimensions of 30 cm×30 cm×10 cm thick. The film was oriented parallel to the beam axis and aligned with the central axis. This phantom contains plastic screws to eliminate air gaps between the film and phantom. Film calibration and analysis was performed using in‐house software and procedure. Film measurements were acquired for both energies (6 MV FFF and 10 MV FFF) for ‘Rails In’ configurations. Film measurements were also acquired with AP delivery (no transmission through couch top and/or rails) to serve as a baseline for surface dose and PDD. The field settings for film measurements were as follows: 500 MU delivered with $5\times 5\;{\text{cm}}^{2}$ field size with delivery to phantom at 100 cm SSD (see Fig. 3 for experimental setup for film measurements).

**Table 1:** Measurement zones for point dose verification measurements

	*Gantry Angle Range*	*Measurement Resolution*	*Type of Incidence to Couch/Rails*
Zone 1	140°−220°	10°	Direct Transmission
Zone 2	100°−130°/230°−260°	2.5°	Oblique Transmission
Zone 3	90°−270°	30°	No Transmission

### D. Lateral shift sensitivity test

The sensitivity of the couch model to lateral displacements was tested. This test was designed to replicate patient motion relative to the couch. The implicit assumption is that the isocenter placement within the patient is correct, but the patient has moved relative to the planned couch position. Using the point dose setup described earlier (see Material & Methods section C), measurements were acquired for various lateral couch displacements with the pinpoint chamber remaining at isocenter. For the 6 MV FFF, “Rails In” geometry, point measurements at 35 posterior gantry angles (90°−270°) were acquired for lateral couch displacements of 2.0 mm, 5.0 mm, and 10.0 mm. For 6 MV FFF “Rails Out” setup, measurements were taken for lateral displacement of 10.0 mm. The 6 MV FFF beam represents the worst‐case scenario for couch displacements because of its larger attenuation through couch components.

## III. RESULTS

### A. Couch component HU value determination

The HU values were assigned to the corresponding couch components as follows: Couch Interior: −930 HU, Couch Surface: −500 HU, Couch Support Rails: 250 HU. These values correspond to the following relative electron densities (where density=1.0 for water): Couch Interior: 0.0842, Couch Surface: 0.4783, Couch Support Rails: 1.099.

### B. Point‐dose verification measurements

Point‐dose measurements were acquired in Solid Water phantom for various combinations of field size, beam energy, and rail positions. The chamber charge readings were converted to dose using the AP field delivery for the corresponding energy and field size. The dose for each measurement point was then scaled according to the $10\times 10\;{\text{cm}}^{2}$ AP field for the corresponding energy to calculate relative dose values. The average magnitude and standard deviation of the percent difference data for ‘Rails In’ configuration is shown in Table 2. The average magnitude of percent difference for ‘Rails Out’ configuration is shown in Table 3

**Figure 4:**
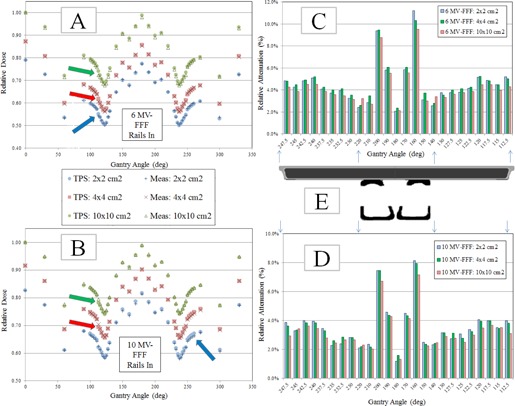
Chamber measurement and TPS calculated dose data for ‘Rails In’ configuration. All charge readings were converted to dose using the AP field for the corresponding energy and field size. The doses were then normalized to the $10\times 10\;{\text{cm}}^{2}$ AP field for the corresponding energy. Arrows represent the maximum deviation for the corresponding field size. Comparison of TPS calculated and measured doses for (a) 6 MV FFF and (b) 10 MV FFF for three field sizes. Measured relative attenuation values for (c) 6 MV FFF (the attenuation was calculated using the opposed gantry angle (180° of separation) that was not transmitted through the couch) and (d) for 10 MV FFF for three field sizes. Schematic (e) of couch showing the couch top and rails. The couch components have been scaled laterally to indicate the presence of a given couch component in the attenuation charts. The arrows indicate that the schematic applies to both the 6 MV FFF chart (above the schematic) and 10 MV FFF chart (below the schematic).

**Figure 5:**
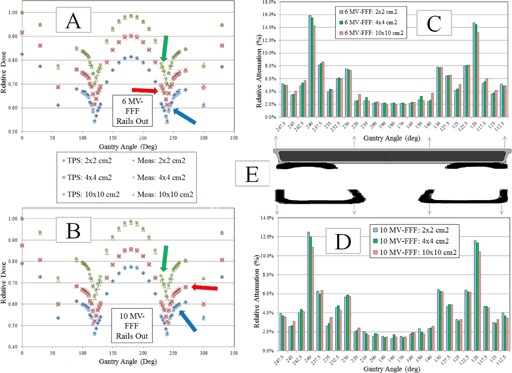
Chamber measurement and TPS calculated dose data for ‘Rails Out’ configuration. All charge readings were converted to dose using the AP field for the corresponding energy and field size. The doses were then normalized to the $10\times 10\;{\text{cm}}^{2}$ AP field for the corresponding energy. Arrows represent the maximum deviation for the corresponding field size. Comparison of TPS calculated and measured doses for 6 MV FFF (a) and for 10 MV FFF, for three field sizes. Measured relative attenuation values (c) for 6 MV FFF (the attenuation was calculated using the opposed gantry angle (180° of separation) that was not transmitted through the couch) and (d) 10 MV FFF for the three field sizes. Schematic (e) of couch showing the couch top and rails. The couch components have been scaled laterally to indicate the presence of a given couch component in the attenuation charts. The arrows indicate that the dimensions of the schematic apply to both the 6 MV FFF chart (above the schematic) and 10 MV FFF chart (below the schematic).

The chamber measurement data are also shown in Fig. 4 (‘Rails In’) and Fig. 5 (‘Rails Out’). The maximum deviation between planned and calculated doses for ‘Rails In’ configuration was: 2.67% (6 MV FFF, $2\times 2\;{\text{cm}}^{2}$ field size), 2.38% (6 MV FFF, $4\times 4\;{\text{cm}}^{2}$ field size), 2.74% (6 MV FFF, $10\times 10\;{\text{cm}}^{2}$ field size), 2.30% (10 MV FFF, $2\times 2\;{\text{cm}}^{2}$ field size), 1.88% (10 MV FFF, $4\times 4\;{\text{cm}}^{2}$ field size), and 2.25% (10 MV FFF, $10\times 10\;{\text{cm}}^{2}$ field size). For ‘Rails Out’ configuration, the maximum deviation between planned and calculated doses was: 2.53% (6 MV FFF, $2\times 2\;{\text{cm}}^{2}$ field size), 1.73% (6 MV FFF, $4\times 4\;{\text{cm}}^{2}$ field size), 3.24% (6 MV FFF, $10\times 10\;{\text{cm}}^{2}$ field size), 2.30% (10 MV FFF, $2\times 2\;{\text{cm}}^{2}$ field size), 1.8% (10 MV FFF, $4\times 4\;{\text{cm}}^{2}$ field size), and 1.68% (10 MV FFF, $10\times 10\;{\text{cm}}^{2}$ field size).

The chamber measurement data were also used to calculate the attenuation of the couch components. To calculate the attenuation, the chamber dose through the couch at a given gantry angle was divided by the chamber dose at the opposed gantry angle (180° of separation). The difference between this ratio and unity is the relative attenuation. The relative attenuation data are shown in Fig. 4 (‘Rails In’) and Fig. 5 (‘Rails Out’).

**Table 2:** Chamber measurement data for ‘Rails In’: average magnitude (along with standard deviation and maximum deviation) of the percent difference between the TPS dose with couch model and the measured dose

	*6 MV FFF*			*10 MV FFF*		
*Rails In*	$2\times 2\;{\text{cm}}^{2}$	$4\times 4\;{\text{cm}}^{2}$	$10\times 10\;{\text{cm}}^{2}$	$2\times 2\;{\text{cm}}^{2}$	$4\times 4\;{\text{cm}}^{2}$	$10\times 10\;{\text{cm}}^{2}$
All Zones	0.90%	0.68%	0.99%	1.09%	0.59%	0.66%
(SD)	(0.72%)	(0.59%)	(0.74%)	(0.64%)	(0.54%)	(0.51%)
[max. dev.]	[1.70%]	[1.96%]	[2.37%]	[1.40%]	[1.36%]	[1.49%]
Zone 1	0.69%	0.74%	1.29%	0.81%	0.45%	0.81%
	(0.71%)	(0.76%)	(0.78%)	(0.36%)	(0.61%)	(0.68%)
	[0.65%]	[0.55%]	[1.04%]	[0.93%]	[0.41%]	[0.55%]
Zone 2	1.11%	0.77%	1.03%	1.32%	0.67%	0.66%
	(0.70%)	(0.55%)	(0.75%)	(0.64%)	(0.49%)	(0.48%)
	[1.70%]	[1.96%]	[2.37%]	[1.40%]	[1.36%]	[1.49%]
Zone 3	0.46%	0.29%	0.49%	0.58%	0.47%	0.43%
	(0.64%)	(0.38%)	(0.39%)	(0.56%)	(0.69%)	(0.28%)
	[1.02%]	[0.58%]	[0.96%]	[0.92%]	[0.65%]	[0.80%]

**Table 3:** Chamber measurement data for ‘Rails Out’: average magnitude (along with standard deviation and maximum deviation) of the percent difference between the TPS dose with couch model and the measured dose

	*6 MV FFF*			*10 MV FFF*		
*Rails Out*	$2\times 2\;{\text{cm}}^{2}$	$4\times 4\;{\text{cm}}^{2}$	$10\times 10\;{\text{cm}}^{2}$	$2\times 2\;{\text{cm}}^{2}$	$4\times 4\;{\text{cm}}^{2}$	$10\times 10\;{\text{cm}}^{2}$
All Zones	0.88%	0.76%	1.05%	0.86%	0.58%	0.63%
(SD)	(0.80%)	(0.51%)	(0.85%)	(0.74%)	(0.38%)	(0.53%)
[max. dev.]	[2.53%]	[1.73%]	[3.24%]	[2.30%]	[1.41%]	[1.68%]
Zone 1	0.28%	0.37%	0.82%	0.25%	0.45%	0.43%
	(0.27%)	(0.32%)	(0.31%)	(0.13%)	(0.17%)	(0.21%)
	[0.65%]	[0.79%]	[1.39%]	[0.39%]	[0.66%]	[0.75%]
Zone 2	1.21%	0.98%	1.26%	1.20%	0.68%	0.75%
	(0.78%)	(0.43%)	(0.98%)	(0.71%)	(0.35%)	(0.59%)
	[2.53%]	[1.73%]	[3.24%]	[2.30%]	[1.41%]	[1.68%]
Zone 3	0.43%	0.43%	0.59%	0.38%	0.39%	0.45%
	(0.70%)	(0.52%)	(0.51%)	(0.54%)	(0.58%)	(0.52%)
	[1.86%]	[1.44%]	[1.43%]	[1.43%]	[1.37%]	[1.37%]

### C. Film verification measurements

**Figure 6:**
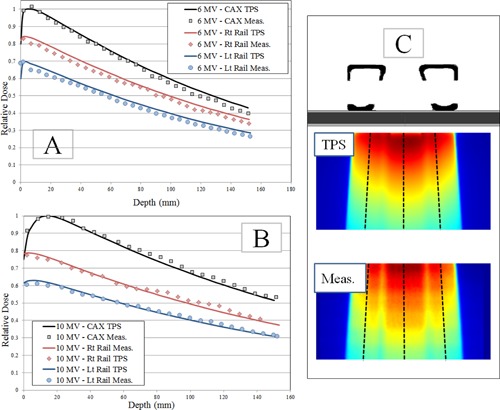
PDD‐style film measurement results with ‘Rails In’ configuration. The film measurement was compared to TPS dose profile in three regions: 1) central axis, 2) transmission through right rail, and 3) transmission through left rail. A scaling factor of 0.8 has been applied to the calculated and measured PDD for the left rail for better visualization of the left and right rail data. Without this scaling factor, the left rail data is similar to the right rail data, and the datasets appear to be superimposed on each other. PDD data for (a) 6 MV FFF and (b) 10 MV FFF. Diagram (c) showing the rays used for PDD calculation on the TPS and film datasets, including a schematic of the couch and to indicate areas of the film that are impacted by the support rails.

**Figure 7:**
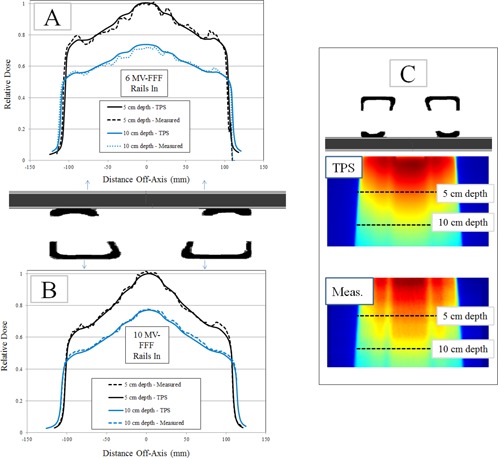
Film profile measurement results for ‘Rails In’ configuration. Schematic of couch is shown to indicate positions of couch components for film measurements. Profile results for (a) 6 MV FFF and (b) 10 MV FFF. Diagram (c) showing film plane and profile depth. The profiles were taken at 5 cm depth and 10 cm depth.

Measurements were acquired with Gafchromic EBT3 film along the central axis of the beam. The film measurement results for 6 MV FFF and 10 MV FFF with ‘Rails In’ configuration are shown in Fig. 6. Crossline profiles were generated from the Gafchromic film measurements; these measurements are shown in Fig. 7. The overall agreement between TPS‐calculated doses and measured film doses was assessed for the PDD (Table 4) and profile measurements (Table 5).

**Figure 8:**
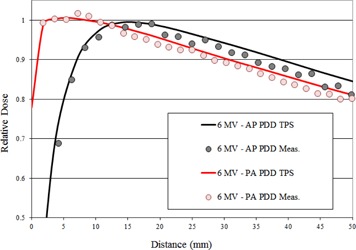
Film measurements for 6 MV FFF for PA delivery (through support structure of couch) and AP delivery (no couch transmission). The curves show the shift in PDD toward the surface due to the presence of the couch, which is also predicted by the TPS calculated dose.

Film measurements were also acquired with AP delivery to serve as a baseline for the couch's effect on superficial dose and dose falloff with depth; these results for 6 MV FFF are shown in Fig. 8. The transmission of the treatment beam through the couch components for PA delivery (gantry 180°) results in an upstream shift of the maximum dose along the central axis; this shift was approximately 10.5 mm for 6 MV FFF and 5.5 mm for 10 MV FFF. The presence of the couch components in the treatment beam also resulted in an increased dose at the surface. For 6 MV FFF, the surface dose increased from 30% with no couch to 80% with PA delivery through couch and support rails. For 10 MV FFF, the surface dose increased from 25% with no couch to 75% with PA delivery through couch and support rails.

**Table 4:** Average magnitude of percent differences for PDD measurements. The PDD measurements are grouped laterally into central axis, left rail, and right rail regions. For each region, the overall agreement (all depths), 0–5 cm depth, and 5–15 cm depth are reported

*% Diff. (PDD)*	*Region*	*Overall*	*0–5 cm Depth*	*5–15 cm Depth*
6 MV FFF	Central Axis	2.98%	1.13%	4.11%
	Rt. Rail	3.38%	3.65%	4.19%
	Lt. Rail	4.83%	3.50%	5.64%
10 MV FFF	Central Axis	2.45%	1.12%	3.11%
	Rt. Rail	2.73%	1.27%	3.42%
	Lt. Rail	2.05%	1.93%	2.10%

**Table 5:** Average magnitude of percent differences for profile measurements. The results are given for two depths of measurement: 5 cm and 10 cm depth. For each depth, the overall agreement, central region of the profile (between the rails; no transmission through rails), and rails (region where beam is transmitted through the rails) data are reported

*% Di*	*ff. (Profiles)*	*5 cm depth*	*10 cm depth*
6 MV FFF	Overall	1.81%	2.31%
	Central (No Rails)	0.71%	2.74%
	Rails	2.19%	2.15%
10 MV FFF	Overall	1.20%	1.67%
	Central (No Rails)	1.04%	0.99%
	Rails	1.27%	1.97%

### D. Sensitivity test

**Figure 9:**
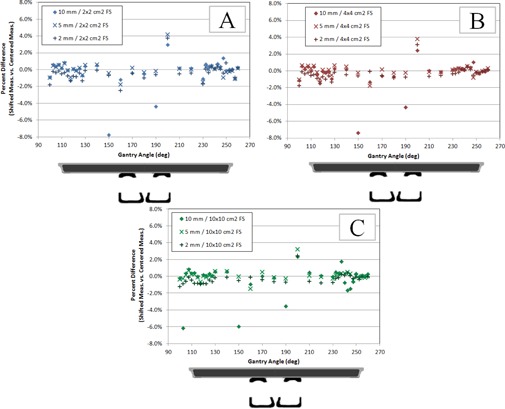
Sensitivity test results for lateral displacements as a function of gantry angle: (a) $2\times 2\;{\text{cm}}^{2}$ field size, (b) $4\times 4\;{\text{cm}}^{2}$ field size, and (c) $10\times 10\;{\text{cm}}^{2}$ field size. A schematic of the couch components is found below each plot to give indication of the geometry.

The sensitivity of couch displacement relative to the target was performed for lateral displacements of 2 mm, 5 mm, and 10 mm. To quantify the sensitivity, the measured value with displacement was compared to the measured value without displacement. The sensitivity test was performed for all posterior gantry angles in the preceding test, covering gantry angles from 90°–270° for a total of 35 discrete gantry angle measurement points. The results for each field size are shown in Fig. 9. The largest sensitivity to lateral shifts occurred at the lateral boundary of the rail structures.

The mean magnitude (standard deviation) of the deviation between shifted and centered measurements over all field sizes tested was 0.61% (0.61%) for 2 mm shifts, 0.46% (0.67%) for 5 mm shifts, and 0.86% (1.46%) for 10 mm shifts (see Fig. 9). For displacements of 2 mm, better than 3.0% agreement was achieved between shifted and centered measurements for 98.1% of measurements with 2 mm displacement, for 97.1% of measurements with 5 mm displacement, and 93.3% of measurements with 10 mm displacement. For displacements of 2 mm and 5 mm, all measurements were within 5.0% of the nominal value. For displacements of 10 mm, 96.2% of measurements (101 out of 105 total measurements) were within 5.0% of the nominal value. The maximum deviation observed for 2 mm shifts was 3.73%, which occurred for $2\times 2\;{\text{cm}}^{2}$ field size at 200° gantry angle. The maximum deviation observed for 5 mm shifts was 4.16%, which occurred for $2\times 2\;{\text{cm}}^{2}$ field size at 200° gantry angle. The maximum deviation observed for 10 mm shifts was 7.79%, which occurred for $2\times 2\;{\text{cm}}^{2}$ field size at 150° gantry angle.

## IV. DISCUSSION

With the ever‐increasing demand for precision on each component of the radiotherapy process, the use of a TPS couch model is a logical and legitimate next step to bring into closer agreement the expected (calculated) dose and delivered dose to the patient. As was stated in the recently published AAPM Task Group 176 (TG‐176) report on the subject, the effect of the support structure on the delivered dose to the patient is a complex mix of increased surface dose, decrease tumor dose, and changes in overall dose distribution. The complexity of the effect warrants the use of a three‐dimensional model, since correction factor‐based methods cannot account for all three effects simultaneously. This study detailed the clinical implementation of a three‐dimensional TPS couch top model, from development to verification testing, for a dedicated stereotactic linear accelerator. Additionally, all measurements for the current study were made for flattening filter‐free treatment beams; we believe this is the first study of its kind to investigate couch modeling and dosimetric effects with FFF beams.

The Varian Edge linac was originally installed at our clinic with FFF beams alone (6 MV FFF and 10 MV FFF). To help with overall clinical flow, however, the 6 MV flattened beam was also installed. Though the couch model verification data for 6 MV flat is not reported in this study, we found the agreement between the TPS calculated doses and measured doses to be similar for 6 MV flat and 6 MV FFF. The maximum discrepancy for each field size was within 0.35% between the two beam modes, and the average discrepancy was within 0.15% for the two beam modes. Overall, we didn't notice any large differences in dosimetric analyses between the flattened and FFF beam modes. To minimize chamber dose measurement uncertainties, we used the AP field delivery for each energy/field size combination and equated the AP field delivery measurement to the AP field calculated dose. In doing so, we were able to minimize the uncertainty associated with FFF chamber dose calibration factor and its relation to the TPS calculated dose. In addition, the AP field calibration minimized uncertainty associated with linac output stability, as all measurements occurred within 15 min of the AP calibration field delivery and linac output changes within this time span are minimal. The localization of the chamber at isocenter (and gantry mechanical isocenter) is another source of uncertainty. We localized the chamber with kV imaging before each measurement session, and our clinical end‐to‐end tests have found the average (SD) gantry isocentricity to be 0.43 mm (0.19 mm). The chamber dose results were compiled over three measurement sessions; the reproducibility of these measurements for all zones with ‘Rails In’ was (on average) 0.38% for 6 MVFFF and 0.45% for 10 MV FFF. There are also several sources of uncertainty for the film dosimetric measurements, including those associated with film uniformity, scanner uncertainties, background, film handling, and registration between film and calculated dose planes. Our clinic utilizes a robust protocol for film processing, scanning, and analysis, which limits the uncertainty for film measurements to approximately 2%.

Other studies have investigated the impact of the couch on the delivered dose distribution. The majority of studies have looked at single‐beam‐angle delivery and have utilized a variety of measurement devices, including cylindrical ion chamber, parallel plate chamber, film, EPID, ion chamber array, and so on. These studies have found that carbon fiber tabletops attenuate the 6 MV beam (on average) in the range of 1.0%–5.0% with posterior normal incidence. In this study, we found that the Qfix kVue Calypso‐compatible couch top attenuated the 6 MV FFF beam on average by about 2.08% for the range of field sizes studied; this value falls within the range of attenuation values reported in TG‐176. This value also agrees with the attenuation value of 2.1% for the Qfix kVue standard couch top, as reported by Seppala and Kulmala^(22)^ for 6 MV beam energy. These studies provide a good baseline for transmission through the couch top for normal beam incidence, but cannot be applied directly to cases when beams traverse the couch at oblique angles.

Some of the aforementioned studies have investigated the dosimetric effects of the couch top for a variety of beam angles and/or delivery types. Mihaylov et al.^(17)^ detailed the implementation of couch top model and subsequent measurement verification for the ExacTrac IGRT couch top. They found that the largest beam attenuation occurred at beam entry angles of about 75° from normal incidence. McCormack et al.^(23)^ found the beam attenuation of a carbon fiber couch insert on the Elekta SLi linac ranged from 2% (normal incidence) to a maximum of approximately 9% (incidence at angle of 70° from vertical). Poppe et al.^(24)^ reported the attenuation of a carbon fiber tabletop for two energies: 1) 6 MV: 2.7% for 180° gantry angle and 3.2% for 150° gantry angle, and 2) 10 MV: 2.3% for 180° gantry angle and 2.4% for 150° gantry angle. Munjal et al.^(25)^ found the maximum attenuation of approximately 3.0% for a 6 MV beam through a carbon fiber couch with tennis racket insert to occur at oblique gantry angles (120°–160° and 240°–200°). Additionally, Seppala et al.^(22)^ found that the maximum attenuation for the Qfix kVue standard couch top of approximately 5.0% occurred at beam angles 60° from the normal 120° gantry angle. Our results with ‘Rails In’ configuration exhibit similar behavior, with the maximum couch attenuation from couch support structure alone occurring at about 60° from normal incidence with an attenuation of approximately 5.0%.

**Figure 10:**
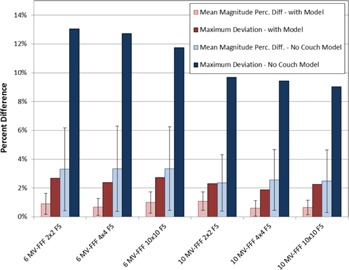
Comparison of chamber data with, and without, the couch model for dose calculation. Error bars represent 1 SD in mean magnitude. The mean magnitude in percent difference with no couch model is approximately 2.5% larger for 6 MV FFF and 2.0% larger for 10 MV FFF. The maximum percent difference with no couch model was as high as 13.0%.

The couch rails, however, provide even further attenuation of the treatment beam. We found that direct PA beam transmission through the rails can provide an additional attenuation of the 6 MV FFF treatment beam by as much as 8.5%. The kVue rails are of different construction than the Varian Exact support structure rails, which were included in the investigation by van Prooijen et al.^(26)^ They found that the Exact rails could attenuate the beam by approximately 10.0%–12.0%. The kVue rail structure was designed to utilize less material (and, therefore, produce less imaging artifact),^(21)^ so the reduction in attenuation is to be expected. If the couch is not modeled, then the error in dose calculation can be substantial. In fact, we found that the mean magnitude of difference between chamber measurements and expected dose with no couch model was larger than the maximum difference between measured and expected dose calculated with the couch model in place (See Fig. 10). Furthermore, the maximum deviation for individual measured points could be as high as 13.0% with no couch model; these regions of largest discrepancy occurred with beams traversing the rails. These results underscore the need for couch modeling, particularly in the context of a specialized linear accelerator developed for stereotactic therapies.

**Figure 11:**
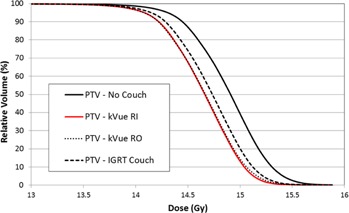
DVH comparison for representative spine SRS VMAT patient with various couch models.

The process of implementing the couch model in Eclipse TPS was described by Wagner and Vorkwerk;^(27)^ their study used a CT scan of the couch to verify optimized HU values after the model was created. For our study, we prospectively acquired a CT scan of the couch top and support rails and used this image set to generate the TPS couch model; perhaps because of this flow in the process, the overall mean error of the chamber measurements was approximately nil and we did not need to optimize the HU value of the couch components. Several studies have investigated the impact of the couch top model on the overall calculated dose for the patient plan for various treatment delivery strategies. Pulliam et al.^(28)^ found that the Varian Exact couch top rails could affect the target dose coverage by approximately 2.0%–5.0% for IMRT and VMAT deliveries for prostate treatments. For this study, a group of four typical SRS spine VMAT plans were calculated using several variations of couch model: 1) no couch model, 2) kVue couch model with ‘Rails In’, 3) kVue couch model with ‘Rails Out’, and 4) Varian IGRT carbon fiber couch top model. For these plans, three plans utilized 6 MV FFF beam energy and 1400 MU/min maximum dose rate, while one plan utilized 10 MV FFF beam energy and 2400 MU/min maximum dose rate. All of these plans utilized two full arcs (gantry rotation from 181° to 179° and vice versa) with complementary collimator angles of 30° and 330°. In particular, no avoidance of couch structures was used, and the gantry and MLC motion were the same for all plans for a given patient. For dose comparison, the PTVD99, PTVD95, Spinal Cord max dose, Spinal Cord D(0.35 cc), Skin D(10 cc), and Skin D(0.03 cc) were compared. The contouring guidelines for these patients were as follows: 1) the PTV structure was delineated according to recommendations of RTOG 0631; 2) the superior/inferior extent of the spinal cord contour was 6 mm superior to the PTV and 6 mm inferior to the PTV; 3) the skin was defined as a 5 mm inner rind from the external contour. The mean volume (SD) for each of the contoured structures was — PTV: 35.1 cc (19.9 cc), Spinal Cord: 2.8 cc (2.3 cc), Skin: 2925.8 cc (1260.4 cc), and Body: 42361.3 cc (15110.3 cc). Normal tissue constraints were chosen to fall in line with recommended tissue constraints from RTOG 0631. The dose comparison data can be found in Table 6. For these patients, the presence of the couch tended to increase surface dose while decreasing dose coverage of the target. Our institution has reported cases of skin dermatitis after spine SRS treatment; the couch top can increase the dose to 10 cc of skin by over 5.5% and to 0.03 cc of skin by over 3.5%. These spine SRS targets are rather small relative to conventional external beam targets. As Higgins et al.^(29)^ showed, the increase in surface dose can depend on field size and is more dramatic for $10\times 10\;{\text{cm}}^{2}$ field size, as compared with the behavior for $40\times 40\;{\text{cm}}^{2}$ field size. For this reason, it is important that the couch's impact for each disease site be thoroughly evaluated. The couch top effect on the target dose coverage was less severe, falling in the range of approximately 1.5%–2.0% on average. For the spine SRS cases included in this analysis, the kVue couch top had a larger effect on the calculated dose distribution than the IGRT couch top. Additionally, a visual DVH comparison for one representative patient is shown in Fig. 11. For patient treatments, the couch can both attenuate the treatment beam (and thereby impact the delivered dose) and collide with the treatment head. The problem of detecting beam–couch intersection was investigated by Muthuswamy and Lam;^(30)^ a TPS couch model, such as the model of the kVue couch top in this study, is a useful, but incomplete, first‐order look into this problem.

**Table 6:** Dose comparison data for four spine SRS patients. The numerical value represents the average percent difference (for all four patients) in the dose metric relative to the value with no couch model included in the plan. Normal tissue constraints were chosen to fall in line with recommended tissue constraints from RTOG 0631

	*No Couch Model*	*k Vu e RI*	*k Vu e RO*	*IGRT Couch*
PTVD99	‐	−2.02%	−0.71%	−0.51%
PTVD95	‐	−2.04%	−0.68%	−0.49%
Spinal Cord Max	‐	−1.48%	−0.39%	−0.19%
Spinal Cord (0.35 cc)	‐	−2.03%	−0.45%	−0.38%
Skin D(10 cc)	‐	5.77%	5.71%	5.36%
Skin D(0.03 cc)	‐	3.60%	3.31%	2.63%

**Figure 12:**
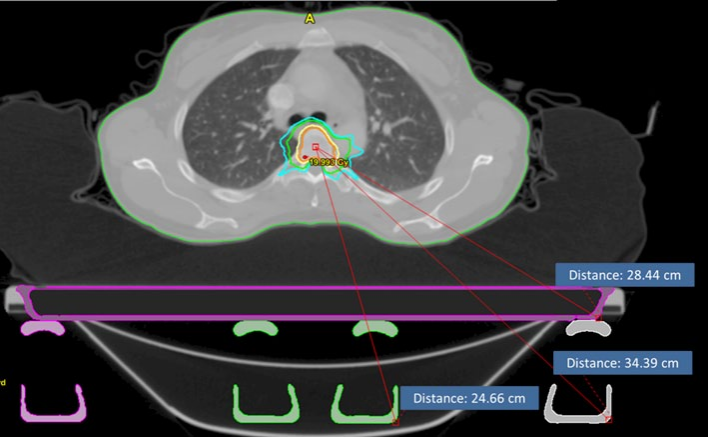
Gantry clearance relative to treatment isocenter for the rails and couch top surface structure for the kVue couch top. In this case, with ‘Rails In’ configuration, the limiting factor for clearance is the couch surface. With ‘Rails Out’ configuration, the limiting factor is the rail structure.

**Figure 13:**
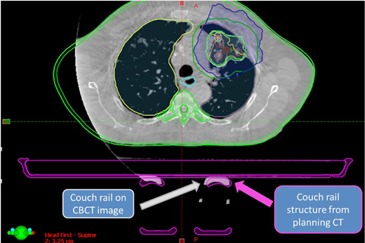
An example of treatment room verification of rail positions. The rail position can be visualized with ‘Rails In’ configuration, and that position can be compared to the couch rail contour from the treatment plan. It should be noted that the rails would be outside the CBCT FOV for ‘Rails Out’ configuration.

The TG‐176 report described several treatment planning strategies to account for the dosimetric effects of the couch top which include modeling of the couch top and beam avoidance. When developing simulation and treatment guidelines in our clinic for the newly installed dedicated stereotactic linear accelerator, it was decided that a combination of both strategies (modeling and avoidance) would be optimal for our clinic. With this in mind, it was decided to treat all patients at our clinic with ‘Rails In’ configuration for several reasons: 1) the use of ‘Rails In’ configuration allows for larger effective gantry clearance relative to the ‘Rails Out’ configuration (see Fig. 12); 2) for ‘Rails In’ configuration, the angles for rail avoidance are less dependent on the isocenter vertical positions relative to the couch; 3) ‘Rails In’ configuration allows for avoidance while maintaining continuous arc delivery; and 4) ‘Rails In’ configuration allows for rail position verification at the treatment unit for cases with CBCT and/or planar kV imaging (see Fig. 13). It is important to note that each clinic must make their own judgments with regard to the simulation and treatment flow for patient treatments. For the case of our clinic in which a heavy stereotactic load, including SRS spine and various SBRT sites, is expected, the priority was placed on developing an accurate model along with a preference for avoidance of the rails with any treatment beams.

## V. CONCLUSIONS

Our study has demonstrated the feasibility of the generation, verification, and implementation of a couch top model for a stereotactic linear accelerator with FFF beams using the TG 176 recommendations. If the couch top is not accounted for in dose calculations, the PTV dose coverage may be overestimated by as much as 2.5% and the errors in calculated dose to organs‐at‐risk may be as high as 5%–10%. The support rails of the couchtop also present difficulties for patient setup with regard to gantry clearance. In our clinic, we elected to treat patients with ‘Rails In’ configuration to maximize the available gantry clearance, minimize oblique beam transmission through the rails, and allow for verification of support rail positions with kV or CBCT imaging. Each clinic must make a decision on the priority levels of various considerations associated with the use of the couch top for treatment planning and dose delivery.

## ACKNOWLEDGMENTS

This research was supported, in part, by a grant from Varian Medical Systems (Palo Alto, CA).

## Supporting information

Supplementary Material

Supplementary Material

Supplementary Material

Supplementary Material
