# Supplementary figures and images for: Generation and verification of QFix kVue Calypso‐compatible couch top model for a dedicated stereotactic linear accelerator with FFF beams
Source: J Appl Clin Med Phys. 2015 Jul 8;16(4):163–80. doi: 10.1120/jacmp.v16i4.5441 (PMC5690015; doi:10.1120/jacmp.v16i4.5441)

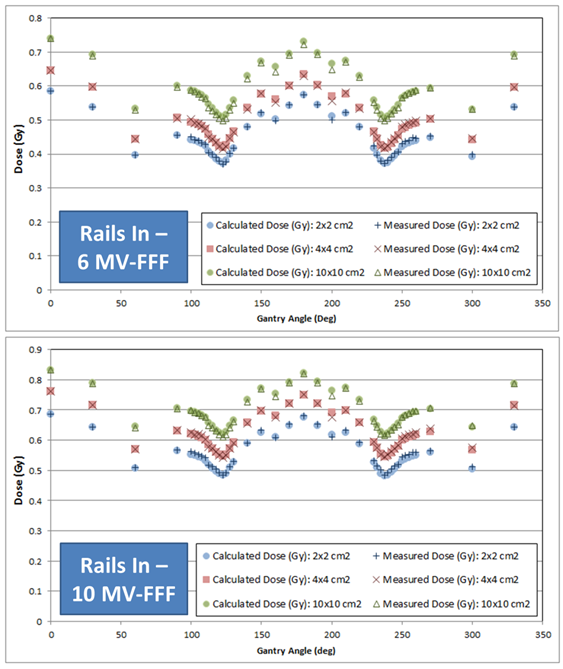

Supplement: Supplementary file 1 — Supplementary Material [file ACM2-16-163-s001.png]

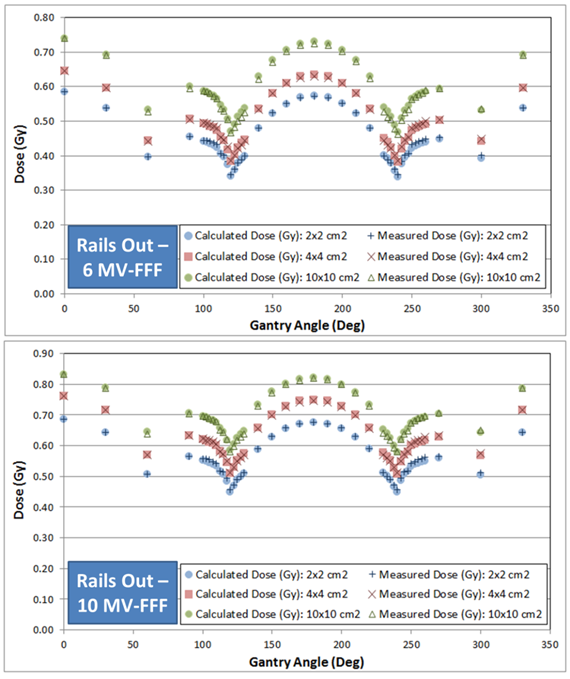

Supplement: Supplementary file 2 — Supplementary Material [file ACM2-16-163-s002.png]

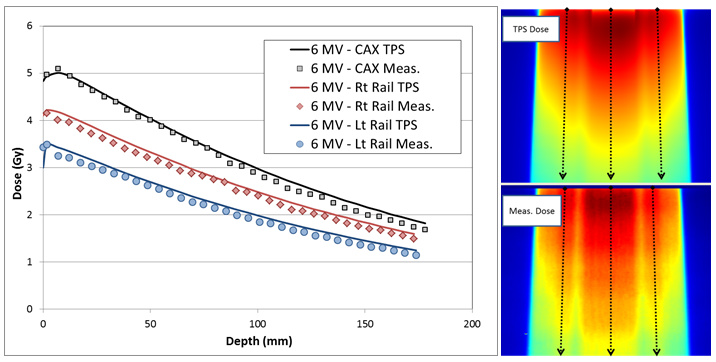

Supplement: Supplementary file 3 — Supplementary Material [file ACM2-16-163-s003.png]

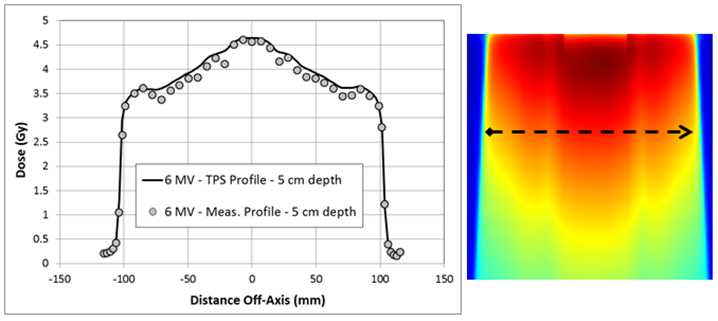

Supplement: Supplementary file 4 — Supplementary Material [file ACM2-16-163-s004.png]
